# 

**Published:** 1888-12

**Authors:** 


					﻿io cts. a copy
DECEMBER. 1888.
$t.oo per year.
WALES*
eJOVRNAL?f
HEAITH
ESTABLISHED 18 J 4
U°i- 35
cl°- IX
OFFICE: 206 BROADWAY. EVENIKCt POST BUTLBTKG. Room 11. M, Y.
Entered as Second-class Matter at the Post-Office, New York.
				

